# Supplementary material for: SwarmGenomics: A Unified Pipeline for Individual‐Based Whole‐Genome Analyses
Source: Mol Ecol Resour. 2026 Mar 19;26(3):e70119. doi: 10.1111/1755-0998.70119 (PMC13000882; doi:10.1111/1755-0998.70119)
Supplement: Supplementary file 1 — Data S1: men70119‐sup‐0001‐DataS1.docx. [file MEN-26-e70119-s001.docx]

FAQs

**Q1: Can I use the pipeline for [X] species?**

SwarmGenomics is currently optimized for diploid species. While it may run on polyploid organisms, this can lead to inaccurate results due to incorrect assumptions about genome structure and heterozygosity.

**Q2: What types of input data does SwarmGenomics accept?**

To run the full pipeline, SwarmGenomics requires a reference genome in FASTA format, ideally high quality and species specific, and raw sequencing reads from a single individual in FASTQ or SRA format. These can come from the same individual or from different individuals of the same or a closely related species.

While both inputs are needed to complete the full workflow, some modules can be run independently with alternative input formats (see Table 1 for details).

**Q3: Is there a recommended sequencing depth or genome size limit for using the pipeline effectively?**

SwarmGenomics works best with sequencing coverage around 20–30× for the diploid individual to ensure reliable results. It can be run on lower coverage data, but results may be less accurate and less reliable. There is no strict genome size limit, though larger genomes will require more computational resources.

**Q4: Why is a virtual machine recommended?**

Many SwarmGenomics modules require substantial computational resources that may exceed the capacity of a typical personal computer, so using a VM on a high-performance server or cluster helps manage these demands efficiently.

**Q5: Do I need internet access throughout the pipeline run?** An internet connection is required to connect to the virtual machine and during software installations. However, smaller operations may be run locally without an internet connection.

**Q6: How do I install the required software for SwarmGenomics?** We recommend following the official installation instructions available in the SwarmGenomics GitHub repository: <https://github.com/AureKylmanen/Swarmgenomics/>.

The repository includes a step-by-step installation guide and optional setup scripts to help you install all required dependencies using conda, download additional tools like the SRA Toolkit and BCFtools, and configure your environment correctly (including for de.NBI VMs).

For best results, please read the instructions carefully and ensure that you have the appropriate system permissions, especially when installing on shared or cluster environments.

**Q7: How to download the reference genome assembly?**

To download a reference genome, we recommend using the NCBI Genome Data page: <https://www.ncbi.nlm.nih.gov/datasets/genome/>.

1. Search for your species of interest.
2. On the species page, select the **FTP** tab to view the available files.
3. Look for the file ending in .genomic.fna.gz — this is the genome assembly file.
4. Copy the link to this file and use the wget command in your terminal to download it. For example:

| $ wget [paste the file link here] |
| --- |

**Q8: How to download raw sequencing reads (in FASTQ or SRA format?)**

To download high-quality raw sequencing reads for whole-genome assembly, we recommend using the NCBI Sequence Read Archive (SRA): <https://www.ncbi.nlm.nih.gov/sra>.

1. Use the **Advanced Search** feature to filter datasets. Recommended search filters include:
   - Platform: Illumina
   - *Source:* DNA
   - Strategy: Genome
   - Layout: PAIRED
   - File Type: FASTQ or SRA
   - Choose a dataset with a large number of reads (e.g., $\geq$10 GB compressed)
2. Once you’ve selected a suitable dataset (e.g., with an accession like SRR12345678), you can download it using sra-tools.

| # To download in SRA format:  prefetch SRRXXXXXXX |
| --- |

Make sure sra-tools is installed and properly configured before running these commands.

**Q9: What does the** “No such file or directory” **error mean, and how can I fix it?**

The “No such file or directory” error in bash means the system can’t find the file or folder you’re trying to access. This often happens because of typos, incorrect file paths, or because the file doesn’t exist in the current directory.

To fix it, check that the filename and path are correct (remember Linux is case-sensitive), use ls to list files in the directory, and verify your current location with pwd.

**Q10: Which parameters need to be changed before running the pipeline?**

Most users only need to modify a small subset of parameters in params.txt before running SwarmGenomics. These typically include:

- **Computational resources:**
  - THREADS — set according to the number of CPU cores available on the system.
- **Working and software directories (required):**
  - WORKING_DIR, TOOL_DIR, and SCRIPTS — update these paths to reflect where the SwarmGenomics project directory, required software, and scripts are located on the local system.
- **Input data paths:**
  - Paths to input FASTQ, BAM, or reference files are automatically constructed using WORKING_DIR and SPECIES. Users should ensure that their input data are placed in the expected directory structure.

**Q11: What if I want to change a parameter and rerun a step?**

To modify an analysis, users can simply edit the relevant value in params.txt (for example, scaffold length cutoffs or allele frequency thresholds) and rerun the corresponding script. The updated parameters are applied automatically, and outputs are regenerated accordingly.

For some steps, such as genome visualisation, rerunning the script will only regenerate plots based on existing intermediate files. If a full recomputation is desired (e.g., recalculating heterozygosity estimates), users may need to remove previously generated intermediate files before rerunning the analysis.

**Q: Still have questions?**

If you have any additional questions or need further assistance, feel free to contact us. We’re happy to help!
